# Network Analysis of Neuropsychiatric Symptoms in Alzheimer’s Disease

**DOI:** 10.21203/rs.3.rs-2852697/v1

**Published:** 2023-04-28

**Authors:** Grace J. Goodwin, Stacey Moeller, Amy Nguyen, Jeffrey L. Cummings, Samantha E. John

**Affiliations:** University of Nevada, Las Vegas

**Keywords:** Alzheimer’s disease, neuropsychiatric, network analysis, MCI, dementia, NPI-Q, agitation, disinhibition, depression

## Abstract

**Background:**

Neuropsychiatric symptoms due to Alzheimer’s disease (AD) and mild cognitive impairment (MCI) can decrease quality of life for patients and increase caregiver burden. Better characterization of neuropsychiatric symptoms and methods of analysis are needed to identify effective treatment targets. The current investigation leveraged the National Alzheimer’s Coordinating Center (NACC) Uniform Data Set (UDS) to examine the network structure of neuropsychiatric symptoms among symptomatic older adults with cognitive impairment.

**Methods:**

The network relationships of behavioral symptoms was estimated from Neuropsychiatric Inventory Questionnaire (NPI-Q) data acquired from 12,494 older adults with MCI and AD during their initial visit. Network analysis provides insight into the relationships among sets of symptoms and allows calculation of the strengths of the relationships. Nodes represented individual NPI-Q symptoms and edges represented the pairwise dependency between symptoms. Node centrality was calculated to determine the relative importance of each symptom in the network.

**Results:**

The analysis showed patterns of connectivity among the symptoms of the NPI-Q. The network (*M*=.28) consisted of mostly positive edges. The strongest edges connected nodes within symptom domain. Disinhibition and agitation/aggression were the most central symptoms in the network. Depression/dysphoria was the most frequently endorsed symptom, but it was not central in the network.

**Conclusions:**

Neuropsychiatric symptoms in MCI and AD are highly comorbid and mutually reinforcing. The presence of disinhibition and agitation/aggression yielded a higher probability of additional neuropsychiatric symptoms. Interventions targeting these symptoms may lead to greater neuropsychiatric symptom improvement overall. Future work will compare neuropsychiatric symptom networks across dementia etiologies, informant relationships, and ethnic/racial groups, and will explore the utility of network analysis as a means of interrogating treatment effects.

## Introduction

Alzheimer’s disease (AD) is the most common cause of dementia in older adults. As of 2022, it is estimated that 6.6 million adults aged 65 and older in the United States are living with AD.^1^ This number is expected to grow to a projected 12.7 million people by 2050. AD is characterized by insidious onset of amnestic symptoms, followed by deterioration of other cognitive abilities and functional independence.^2^ Mild cognitive impairment (MCI) can be considered an early stage of AD progression and is characterized by subtle changes in memory and cognition, while adaptive functions are spared.^3^

In addition to decline in cognitive functioning, patients with MCI and AD exhibit behavioral or neuropsychiatric changes. Neuropsychiatric symptoms (NPS) refer to behavioral, affective, and personality changes that can be attributed to underlying neurodegenerative disease. Common symptoms include apathy, depression, aggression, anxiety, and sleep disturbance, and less common symptoms include irritability, appetite changes, aberrant motor behavior, delusions, disinhibition, euphoria, and hallucinations.^4^ Almost all patients exhibit neuropsychiatric symptoms at some point in their disease,^5–7^ and apathy and depression are the most frequently reported disturbances among patients with AD^5,8^ and MCI.^6^ Neuropsychiatric symptoms are often present in the early clinical stages of neurocognitive decline and are therefore considered diagnostic and prognostic indicators of neurodegenerative disease.^9–14^ The type of symptoms expressed may also indicate underlying pathological changes. For example, Braak staging of neurofibrillary tau burden was tied to increased odds for neuropsychiatric symptoms in those with autopsy-confirmed AD. Specifically, even early subcortical neurofibrillary tangle accumulation was associated with agitation, anxiety, appetite change, depression, and sleep disturbance.^15^ A recent review highlighted the neuroanatomical correlates of NPS in AD and identified symptom-general and symptom-specific patterns of brain pathology. Damage to the anterior cingulate cortex and orbitofrontal cortex were associated with most neuropsychiatric symptoms. Additionally, there is evidence of symptom-specific neurobiological correlates of neuropsychiatric symptoms (e.g., frontal-limbic circuit involvement in depression).^16^

Neuropsychiatric symptoms are distressing for patients and caregivers and are associated with increased functional and cognitive impairment,^4,17–20^, hospitalization, caregiver burden,^21^ and institutionalization.^5,22^ Nonpharmacologic interventions (e.g., environmental modifications, exercise, reminiscence therapies, caregiver training) are considered the first line of management of neuropsychiatric symptoms. However, most patients are eventually treated with psychotropic medications as the disease progresses and symptoms worsen. Evidence of efficacy of nonpharmacologic and pharmacologic interventions is mixed; while some patients and caregivers experience relief from treatment, others do not. Additionally, psychotropic medication agents have side effects, are associated with greater morbidity and mortality, and have limited evidence for efficacy among patients with dementia. Atypical antipsychotic medications, benzodiazepines, and sedative/hypnotic medications are sometimes used to treat a variety of neuropsychiatric symptoms, but may be associated with accelerated cognitive decline, metabolic syndrome, cardiovascular events, and falls.^23^

Assessment of neuropsychiatric symptoms in AD is important for accurate differential diagnosis, disease management, and understanding the neurobiological underpinnings of behavioral changes in dementia.^24^ The Neuropsychiatric Inventory Questionnaire (NPI-Q) is a widely used informant-based questionnaire that assesses the presence and severity of 12 neuropsychiatric symptoms evident within the last month.^25^ Previous studies have used factor analysis, cluster analysis, and latent class analysis to categorize symptoms of the NPI and NPI-Q; however, the taxonomy of neuropsychiatric symptoms in AD remains unclear. There is relatively low concordance among studies attempting to identify neuropsychiatric symptom clusters or domains.^26^ Some studies have identified 3 symptom domains^27–29^ while others have identified 4 or more.^13,19,30^ These item-level and domain-level examinations do not capture symptom complexity, interaction, or comorbidity. One study addressed this by examining comorbidity among neuropsychiatric symptoms among patients with AD by calculating the odds ratio of a given symptom in the presence of another. They identified several statistically significant combinations of symptoms; for example, the odds of endorsing hallucinations were 6.49 times higher in those with delusions than in those without and the odds of endorsing aberrant motor behavior were 9.48 times higher in those with disinhibition versus those without. Their findings highlight complex interrelationships among neuropsychiatric symptoms^29^ and provide an empirical foundation for the classification of neuropsychiatric symptoms in AD. However, these approaches do not consider the co-occurrence of multiple (i.e., more than two) neuropsychiatric symptoms.

Evaluating the meaning of neuropsychiatric symptoms in AD requires further study. While cognitive symptoms in AD may follow a fairly uniform trajectory, neuropsychiatric symptoms vary widely and patterns remain elusive as they rarely align with changes in cognitive function.^18^ Additionally, patient-specific factors contribute to symptom heterogeneity.^8,26^ There is also evidence of clustering and comorbidity among neuropsychiatric symptoms, and symptoms tend not to be experienced in isolation.^5^ It is therefore important that neuropsychiatric symptoms be examined in relationship to one another.

Recently, researchers have posited that network models could provide a detailed characterization of psychological syndromes.^31^ According to network theory, psychological disorders can be viewed as a set of interacting symptoms that amplify, reinforce, and maintain each other.^32–34^ Network analysis highlights clusters of strongly interconnected symptoms and quantifies the relative importance of individual symptoms.^35–36^ Central symptoms, or symptoms with a large number of connections to other symptoms in a network, represent core features of a syndrome^34^, and can, theoretically, be considered targets for widespread symptom reduction.^37^ Network analysis has been used to characterize symptom presentation and progression in schizophrenia,^38^ depression, anxiety,^39^ post-traumatic stress disorder,^40^ and sport-related concussion.^41–43^ A network perspective may be equally illuminating for characterizing neuropsychiatric symptoms in AD. Network analysis could provide unique insights into symptom maintenance and progression and identify central symptoms that may be efficient targets for widespread symptom reduction.

The network structure of neuropsychiatric symptoms in AD has yet to be characterized. The current investigation leveraged the National Alzheimer’s Coordinating Center (NACC) Uniform Data Set (UDS) to examine the network structure of neuropsychiatric symptoms among older adults with cognitive impairment. Given that the severity and nature of initial symptoms consistently predict disease course, data from participants’ initial visit was used. We examined symptoms among patients diagnosed with MCI or dementia due to AD. This study aimed to conceptualize the comorbidity and complexity of neuropsychiatric symptoms in AD and provide a foundation for personalized approaches to symptom management.

## Methods

The NACC UDS is a comprehensive data repository for research on neurodegenerative disorders, including AD. The UDS contains longitudinal data that have been collected since 2005 at NIA-funded Alzheimer’s Disease Research Centers (ADRCs) across the United States. Data elements and collection methods have been described previously.^44–47^ The NACC UDS includes neuropsychological, behavioral, medical, and health history data that is used to accurately diagnose neurodegenerative disease and track its course.^47^ Participant written consent was obtained by each ADRC’s institutional review boards.

### Participants

**Figure 1:**
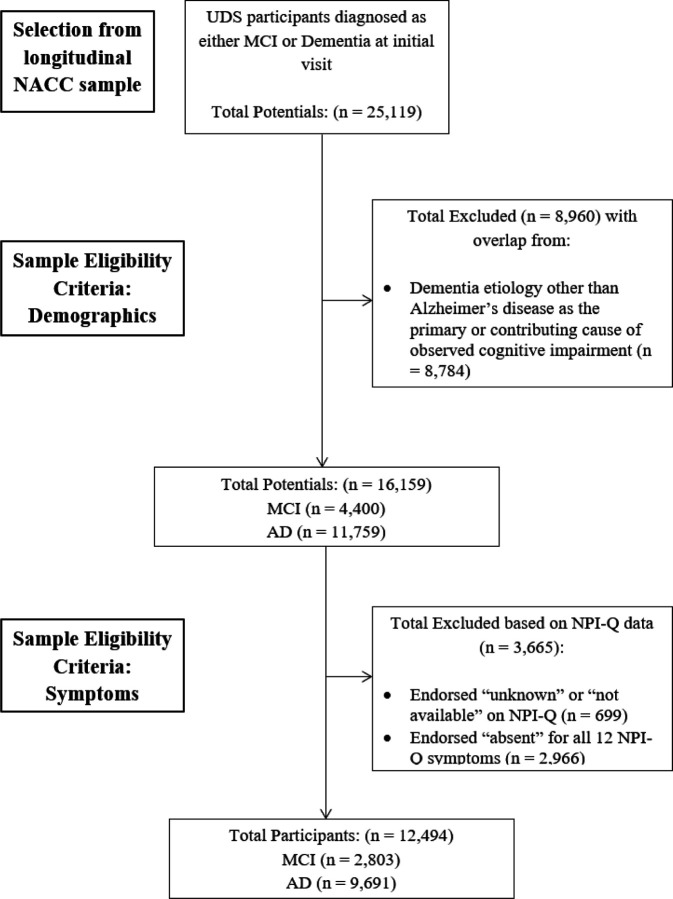
Participant Selection Diagram

Participants were selected from the NACC UDS (v1-v3) data set (https://naccdata.org/). Participant evaluations from initial visits were used in the current analysis and were completed at funded ADRCs during the period between September 2005 and the freeze date of December 2021. Patient demographic variables and diagnostic status were used to identify the sample for analysis (Figure 1). The total sample for all initial participant visits was 44,713. The following inclusion criteria were applied for sample identification: cognitive status of MCI or dementia (n = 25,119); AD was the primary or contributing cause of observed impairment (n = 16,335); participants were 50 years or older (n = 16,159); and at least one symptom on the NPI-Q was endorsed. Participants were excluded if they endorsed “unknown” or “not available” on any NPI-Q items. The final sample (n = 12,494) consisted of older adults (M_age_=73.9, SD_age_=9.37; 46.2% male, 53.8% female, M_education_ = 15.21 years, SD_education_ = 8.58 years) who predominantly identified as non-Hispanic white (74.5% non-Hispanic white, 11% non-Hispanic Black, 8.5% other, 5.8% Hispanic white, .3% Hispanic Black). The majority of the sample met criteria for dementia (77.6% dementia, 22.4% MCI) and AD was the presumed primary etiology in 93.9% and contributing etiology in 6.1%. See tables 1 and 2 for demographic and descriptive data.

### Measures

#### Race and Ethnicity

In order to examine participant race and ethnicity, a new variable was calculated that combined data from the NACC-derived race variable for the six main census race groups and the UDS ethnicity variable for Hispanic/Latino ethnicity. Five new racial/ethnic groups were created from these data: Non-Hispanic white, Hispanic white, Non-Hispanic Black, Hispanic Black, and all other categories.

#### Cognitive Status and Alzheimer’s Disease Status

Cognitive impairment was classified through a variable derived from NACC that includes the following categories: 1) normal cognition, 2) impaired-not-MCI (subjects who are cognitively impaired but do not meet criteria for MCI), 3) MCI (subjects with either amnestic or non-amnestic MCI), and 4) dementia (subjects who have a cognitive diagnosis of dementia).^48^ AD etiology was classified according to variables derived from NACC that includes the following categories: 1) primary (AD is the primary cause of observed cognitive impairment), 2) contributing (AD is a contributing cause of observed cognitive impairment), 3) non-contributing (AD was a non-contributing cause of observed cognitive impairment), 4) cognitively impaired but not AD (no etiological diagnosis of AD), and 5) diagnosis of normal cognition.^47^ Only those with a cognitive diagnosis of MCI or dementia and those with an etiology of AD as a primary or contributing cause of observed impairment were included in the analysis sample.

#### Characterization Variables

The Functional Activities Questionnaire (FAQ) is an informant-report measure of a patient’s ability to perform instrumental activities of daily living (IADLs). Informants rate the extent to which patients require help with ten IADLs over the last four weeks (0 = Normal–3 = Dependent). The Geriatric Depression Scale (GDS) is a self-report measure of depression symptoms.^49^ Patients rate whether or not they experienced 15 depression symptoms over the last week (0 = No, 1 = Yes). Scores are summed and a score of and scores of 9–11 indicate moderate depression and scores of 12–15 indicate severe depression. The Clinical Dementia Rating (CDR) Dementia Staging Instrument is a 5-point scale that characterizes six domains of cognitive and functional abilities.^50^ Information is obtained through semi-structured interview of the patient and informant, and clinicians rate the patient’s level of overall impairment (0.0 = No impairment–3.0 = Severe Impairment).

#### Primary Outcome Measure

The NPI-Q is a widely used measure to assess neuropsychiatric symptoms among clinical populations.^25^ The NPI-Q relies on a caregiver/informant report of the presence and severity of 12 neuropsychiatric symptoms evident within the past month. Assessed symptoms include delusions, hallucinations, agitation/aggression, depression/dysphoria, anxiety, elation/euphoria, apathy/indifference, disinhibition, irritability/lability, motor disturbance, nighttime behaviors, and appetite/eating problems.^25^ Informants endorsed the presence of each symptom (0 = No, 1 = Yes). The total NPI-Q symptom score ranges from 0 to 12. The NPI-Q has adequate psychometric properties, including acceptable test-retest reliability and convergent validity.^25^

### Analyses

#### Network Estimation

Statistical analyses were conducted in R version 4.0.3. using qgraph,^51^, bootnet,^52^ and networktools.^53^ Network analysis allows for the graphical representation of symptoms (nodes) and the statistical relationship among them (edges). Item endorsement on the NPI-Q is dichotomous (i.e., symptoms are either present or absent), so methods that calculate partial correlations between nodes are not appropriate for analysis, given that they require assumptions of linearity and normality.^54^ Instead, a binary equivalent of the Gaussian approximation method was used. The eLasso method, which is based on the Ising model, estimates parameters using logistic regressions.^54^

The network was estimated from individual NPI-Q item scores. Nodes represent the threshold of each NPI-Q symptom, or the independent disposition of that symptom to be present or absent without the influence of neighboring symptoms. Each node is regressed on all other nodes in the network. Edges represent the pairwise dependency between two nodes after controlling for all other nodes in the network. The network represents the conditional probability of an observed binary variable (e.g., presence/absence of delusions) given all other measured variables (e.g., presence/absence of all other NPI-Q symptoms).^54–55^

Two methods were applied to balance network sensitivity and specificity. First, networks were regularized using the recommended least absolute shrinkage and selection operator (LASSO) penalty.^54^ The tuning parameter was chosen using the Extended Bayesian Information Criterion (EBIC^56^). The EBIC hyperparameter gamma value was set to .25, which is recommended for estimating binary networks.^54^ This process removes weak and spurious edges and returns a sparse network in which a small number of likely genuine edges are used to explain network structure.^52^ Second, the “OR-rule” was used to determine the final set of edges. The “OR-rule” requires only one of the two regression coefficients to be nonzero (i.e., for nodes j and k, either b_jk_ or b_kj_ is nonzero) in order for the edge to be retained in the network, thereby increasing the number of estimated connections. Alternatively, a stricter “AND-rule” can be applied, which requires both regression coefficients to be nonzero for the edge to be retained in the network.^54^ The less stringent “OR-rule” was more appropriate in this study given that regularization had already been applied.

Once the final edges were selected, the weighted value of each edge was calculated by taking the mean of both regression coefficients (i.e., for nodes j and k, the average of b_jk_ and b_kj_) for a given pair of nodes. The final network consisted of weighted edges between all node pairs and represented a statistical association between nodes after controlling for all other nodes in the network.^54^ The Fruchterman-Reingold algorithm was used for the graph layout, such that nodes were placed close together if they had stronger or more connections to each other.^54,57^

#### Node Centrality

Centrality was computed to determine a symptoms’ relative importance within the network. Node strength and expected influence measure the number of connections extending from a given node that is weighted by eLasso coefficients.^36,54,58^ Strength is calculated by taking the sum of the absolute value of all edges extending from a given node.^36^ Expected influence considers negative edges and is calculated by taking the sum of all edges extending from a given node.^58^ For both metrics, higher values indicate greater node importance.^36,58^

#### Network Accuracy

Edge-weight accuracy, centrality stability, and edge-weight and centrality difference tests were computed to determine network accuracy.^36^ To measure edge-weight accuracy, nonparametric bootstrapped confidence intervals (CIs, 95%) were constructed around the regularized edge-weights. Large CIs suggest that edge-weights do not significantly differ. To assess centrality stability, a case-dropping subset bootstrap approach was employed. The centrality stability (CS) coefficient signifies the maximum proportion of cases that can be dropped while maintaining a large correlation (r=.70) between the full- and subset-sample networks’ centrality values. CS-coefficients should be above .50 and no lower than .25 for the centrality indices to be trustworthy.^36^ Edge-weight and node centrality differences were examined using calculated difference scores for each pair of bootstrapped edge-weight/centrality. Edge-weights and centralities are considered trustworthy if zero is included in the bootstrapped CI.

## Results

**Figure 2:**
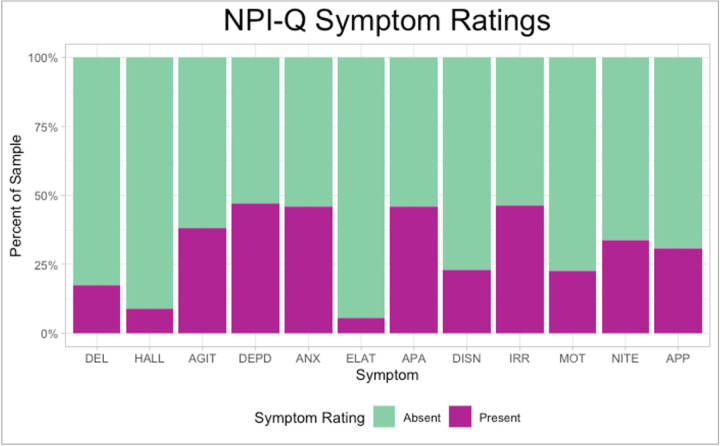
Neuropsychiatric Symptom Frequency *Note.* Percentage of participants who endorsed individual NPI-Q symptoms. Symptom present = NPI-Q item rating of 1. Symptom absent = NPI-Q item rating of 0. “DEL” = Delusions, “HALL” = hallucinations, “AGIT” = agitation/aggression, “DEPD” = depression/dysphoria, “ANX” = anxiety, “ELAT” = elation/euphoria, “APA” = apathy/indifference, “DISN” = disinhibition, “IRR” = irritability/lability, “MOT” = motor disturbance”, “NITE” nighttime behaviors, “APP” appetite/eating problems

On average, 3 or more symptoms were endorsed on the NPI-Q (MCI: *M* = 2.75, *SD* = 1.82, range = 1–12; dementia: *M* = 3.90, *SD* = 2.32, range = 1–12). Symptom severity was mild overall (MCI: *M* = 3.78, *SD* = 3.32; dementia: *M* = 6.05, *SD* = 4.78). The most frequently endorsed symptom was depression/dysphoria (*M* = .7 *SD* = .50), followed closely by anxiety (*M* = .46, *SD* = .50), apathy/indifference (*M* = .46, *SD* = .50), and irritability/lability (*M* = .46, *SD* = .50) (Figure 2). See tables 1 and 2 for additional sample characterization through summary of CDR scores and FAQ scores, respectively.

### Network Architecture

**Figure 3:**
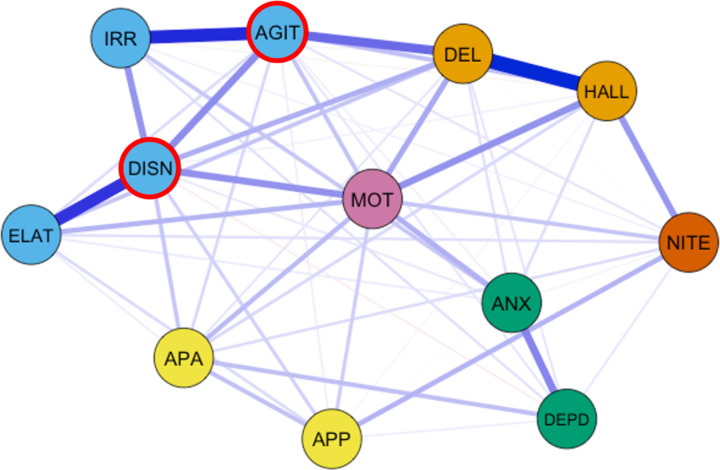
Network of Neuropsychiatric Symptoms. *Note.* The layout of the graph used the Fruchterman-Reingold algorithm. Nodes with highest strength centrality and expected influence are outlined in red. “DEL” = Delusions, “HALL” = hallucinations, “AGIT” = agitation/aggression, “DEPD” = depression/dysphoria, “ANX” = anxiety, “ELAT” = elation/euphoria, “APA” = apathy/indifference, “DISN” = disinhibition, “IRR” = irritability/lability, “MOT” = motor disturbance”, “NITE” nighttime behaviors, “APP” appetite/eating problems.

Out of a possible 66 edges, 57 (86%) were retained (*M*_weight_=.28) following regularization. The network consisted of mostly positive edges (Figure 3). The strongest edges were found between delusions and hallucinations (edge-weight = 1.51), agitation/aggression and irritability/lability (edge-weight = 1.31), elation/euphoria and disinhibition (edge-weight = 1.21), depression/dysphoria and anxiety (edge-weight = .72), agitation/aggression and disinhibition (edge-weight = .68), and disinhibition and irritability/lability (edge-weight = .63). The network also exhibited edges between delusions and agitation/aggression (edge-weight = .83), disinhibition and motor disturbance (edge-weight = .65), hallucinations and motor disturbance (edge-weight = .64), and hallucinations and nighttime behaviors (edge-weight = .61).

**Figure 4:**
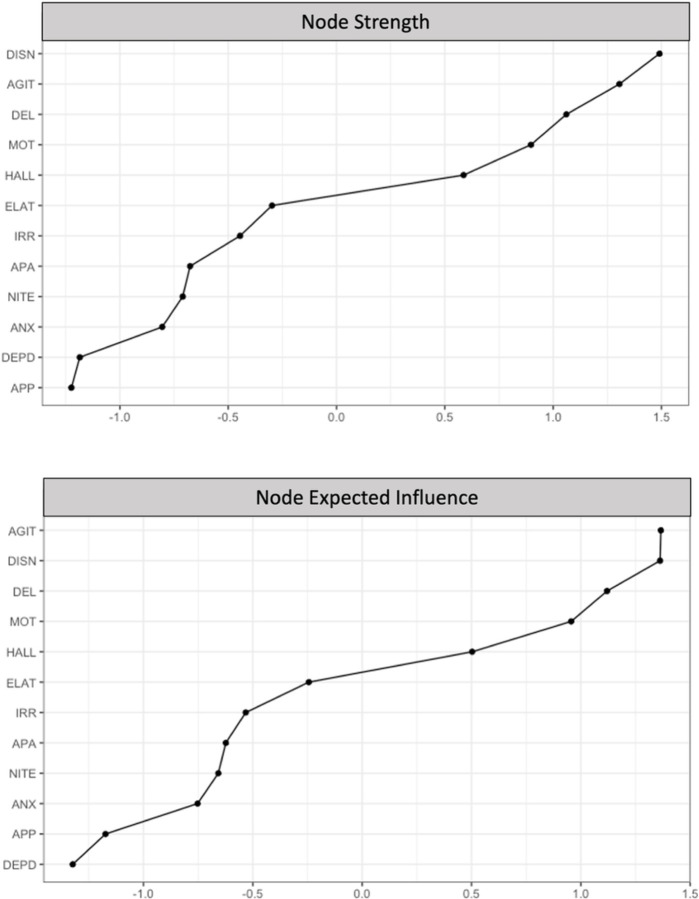
Rank Order of Node Strength and Expected Influence Values *Note.* Rank order of node strength (top graph) and expected influence (bottom graph). Nodes are presented in order from highest (top of figure) to lowest strength (bottom of figure). Expected influence values are shown as standardized *z*-scores.

Node strength (*CS(*cor=.7)=.75) and expected influence (*CS(*cor=.7)=.75) were stable and are interpretable indices of centrality (Supplementary Figure 1). Disinhibition had the highest node strength (*z* = 1.49), and agitation/aggression had the highest expected influence (*z* = 1.37). Disinhibition and agitation/aggression shared most of their connections with other behavioral symptoms, including irritability/lability, elation/euphoria, and motor disturbance. Depression/dysphoria and appetite/eating problems had the lowest node strength and expected influence (Figure 4).

### Network Accuracy

Confidence intervals were wider than optimal around the parameter estimates for edge-weight, suggesting that estimation of edge-weight values should be interpreted with caution (Supplementary Figure 2). While there were considerable overlaps among the edge-weight CIs, there was no overlap around the strongest edges in the network, suggesting that the order of the strongest edges are accurate and interpretable.

Bootstrapped differences tests showed that edge-weight values significantly differed from one another, providing additional evidence that the order of edge-weight values is interpretable (Supplementary Figure 3). Additionally, node centrality values significantly differed from one another, providing additional evidence that the order of centrality values is interpretable (Supplementary Figures 4 and 5). In sum, results suggest that the network was accurate, stable, and interpretable.

## Discussion

The present study used network analysis to examine the associations among neuropsychiatric symptoms occurring in a large sample of symptomatic older adults with cognitive impairment. Neuropsychiatric symptoms become increasingly evident throughout AD progression and are the most likely symptoms to require behavioral and pharmacological intervention.^59^ These symptoms, along with other behavioral symptoms, are difficult to manage, are highly distressing, and confer risk for patients, caregivers, and clinicians.^60^ Moreover, these behaviors are often directed toward or experienced by caregivers, which leads to increased caregiver burden and decreased quality of life.^59,61^

Within our analytic sample, participants had mostly mild (MCI) or moderate (dementia) global impairment. Three or more neuropsychiatric symptoms were endorsed on average, and neuropsychiatric and depression symptom severity were mild overall. Consistent with previous research,^29^ the interconnectedness of symptoms observed in the network suggests that neuropsychiatric symptoms are highly comorbid. While the present model cannot determine causality, results suggest that neuropsychiatric symptoms may be mutually reinforcing, whereby activation of one symptom results in cascading activation of other symptoms throughout the network. For example, disinhibition was associated with motor disturbance, motor disturbance was associated with hallucinations, hallucinations were associated with nighttime behaviors, and nighttime behaviors were associated with appetite and eating problems.

As in previous studies,^6,62^ depression was the most commonly endorsed symptom in the current sample. However, depression was not a highly central symptom in the network. Our results suggest that depression, while common, is not predictive of neuropsychiatric symptoms more broadly. However, given that depression in AD is associated with greater functional and cognitive disability, caregiver burden, and reduced quality of life,^62^ it may be an important standalone symptom to evaluate and ameliorate in this population.

Disinhibition and agitation/aggression emerged as central symptoms in the network, suggesting that they likely influence the activation or persistence of other neuropsychiatric symptoms. Disinhibition refers to difficulty suppressing inappropriate or maladaptive thoughts or behaviors.^63^ Agitation is characterized by physical aggression, verbal aggression, resistance to attempts at care, and hyperactivity. Aggression refers to more marked verbal insults (e.g., shouting, cursing) and physical behaviors (e.g., hitting, kicking, biting, throwing objects). With respect to symptom aggregation, the presence of disinhibition increases the likelihood of all other behavioral symptoms being present and is most strongly linked to agitation. Symptoms with strong relationships to one another within the network, as with irritability and agitation, may reflect strong temporal associations and co-occurrence, as irritability is often a precursor or accompanying feature of agitation/aggression.^64^ Thus, when agitation is present and endorsed, irritability is likely also to have occurred. Although speculative, our observations suggest that the presence of some neuropsychiatric symptoms predicts other neuropsychiatric symptoms.

According to network theory of mental disorders, central symptoms represent core features of a syndrome, and “deactivating” a core symptom could, in turn, deactivate other symptoms within the network.^32^ Thus, treating or managing disinhibition and agitation/aggression may predict alleviation of overall neuropsychiatric symptoms. In sum, our findings lend further support to the importance of these network relationships as key features of neuropsychiatric symptoms in AD.

### Limitations and Future Directions

Given the cross-sectional design of this study, we cannot infer temporal precedence between symptoms. It is important that future research continues to explore patterns across the disease course among neuropsychiatric symptoms to better identify conversion risk and determine whether neuropsychiatric symptom networks change as disease progresses. Additionally, while central symptoms can be considered theoretical targets for reducing associations among other symptoms, treatment simulation studies are mixed^65^ and empirical data are needed. This work should be replicated in NPI-Q networks of patients before and after intervention to determine the extent to which other neuropsychiatric symptoms are reduced when central symptoms are removed or ameliorated.

The NPI-Q is an informant-based measure and symptoms can be misinterpreted, underreported, or overreported. Further, the NPI-Q asks informants to endorse symptoms only if they have occurred in the past month, which does not consider fluctuating disease presentations. Network relationships should be studied using patient or clinician reports to determine if network structure persists across different informant relationships (e.g., spousal caregivers vs. siblings vs. children) and characteristics (e.g., time spent with participant and/or residential setting). NPI-Q symptom descriptions may be subject to cultural bias wherein the informant does not acknowledge or interpret the symptom as part of the disease. Relatedly, ethnic and racial differences in neuropsychiatric symptomatology remain understudied and should be addressed in future work. While our analyses incorporated data from a diverse ethnic and racial cohort, future analyses will examine these relationships more intentionally. Finally, examining the extent to which pre-morbid, environmental, and sociodemographic factors may moderate the interrelationships among neuropsychiatric symptoms could better characterize symptom heterogeneity. Areas for future research may center on associations of neuropsychiatric symptom clusters with other markers of disease, such as apolipoprotein E genotype, cerebrospinal fluid biomarkers, and amyloid and tau positron emission tomography.

## Conclusions

In summary, this study examined the network structure of neuropsychiatric symptoms occurring among older adults with MCI and AD dementia. Results quantify the relationships between symptom pairs and identify highly influential symptoms in the network. Our findings highlight neuropsychiatric symptom comorbidity and suggest that disinhibition and agitation/aggression may be important targets for intervention. A network perspective may improve current understanding of neuropsychiatric symptomatology in this population. Future research is needed to determine the clinical utility of network models in assessment and treatment.

## Supplementary Material

Supplement 1

## Figures and Tables

**Table 1.** Participant Demographics Stratified by Cognitive Status

Participant Demographics Stratified by Cognitive Status (*N =* 12,494)		
	MCI	Dementia
*n*	2803	9691
Age [M(*SD*)]	73*(8.23)*	73.9*(9.56)*
Sex		
Male	50.48%	44.98%
Female	49.52%	55.02%
Education Years [M(*SD*)]	16*(6.81)*	15*(9.01)*
Ethnic Racial Group (%)		
non-Hispanic white	75.10%	74.26%
non-Hispanic Black	10.49%	11.09%
Hispanic white	5.42%	5.91%
Hispanic Black	0.32%	0.24%
Other	8.67%	8.49%
Alzheimer's Disease Etiology		
Primary Etiology	94.18%	93.81%
Contributing Etiology	5.82%	6.19%
CDR Global Impairment rating (%)		
None (0.0)	3.28%	0.29%
Questionable (0.5)	94.40%	28.99%
Mild (1.0)	2.32%	45.73%
Moderate (2.0)	0.00%	17.19%
Severe (3.0)	0.00%	7.80%
GDS Total Score[M(*SD*)]	2.64*(2.57)*	2.73*(2.72)*

*Note.* M = mean, SD = standard deviation. MCI = mild cognitive impairment. CDR = clinical dementia rating. GDS = geriatric depression scale. MCI vs. Dementia derived from Cognitive Status at UDS Visit variable. Alzheimer’s disease etiology derived from clinician diagnosis of cause of observed cognitive impairment due to Alzheimer’s disease. Impairment ratings derived from the Clinical Dementia Rating Global Impairment score. Depression derived from Geriatric Depression Scale total score.

**Table 2.** Frequency of Functional Impairment

Functional Activity	Normal	Has difficulty, does by self	Requires assistance	Dependent	Unknown/Not Applicable
Bills	17.93%	12.97%	15.77%	41.24%	12.10%
Taxes	14.30%	9.68%	14.97%	44.14%	16.92%
Shopping	30.61%	19.04%	21.31%	25.72%	3.31%
Games	31.63%	22.07%	14.51%	18.33%	13.47%
Stove	54.20%	16.66%	9.37%	16.71%	3.05%
Meal Prep	31.48%	14.81%	14.60%	26.05%	13.06%
Events	32.38%	25.48%	20.41%	19.57%	2.17%
Pay Attention	38.16%	30.67%	17.91%	11.97%	1.29%
Remembering Dates	14.95%	22.20%	31.13%	30.75%	0.98%
Travel	22.91%	19.02%	16.42%	39.68%	1.98%

*Note.* Impairment ratings derived from the Functional Activities Questionnaire (0 = Normal; 1 = Has difficulty, does by self; 2 = Requires assistance; 3 = Dependent; 8,9= Not applicable or unknown.

## Abbreviations

AD: Alzheimer’s disease
MCI: Mild cognitive impairment
NPS: Neuropsychiatric symptoms
NPI-Q: Neuropsychiatric Inventory Questionnaire
NPI: Neuropsychiatric Inventory
NACC: National Alzheimer’s Coordinating Center
UDS: Uniform Data Set
NIA: National Institute on Aging
ADRC: Alzheimer’s Disease Research Center
FAQ: Functional Activities Questionnaire
IADL: Instrumental activities of daily living
GDS: Geriatric Depression Scale
CDR: Clinical Dementia Rating
EBIC: Extended Bayesian information criterion
CI: Confidence interval
CS: Centrality stability
