# Supplementary material for: Network Analysis of Neuropsychiatric Symptoms in Alzheimer’s Disease
Source: Res Sq. 2023 Apr 28:rs.3.rs-2852697. Preprint. [Version 1] doi: 10.21203/rs.3.rs-2852697/v1 (PMC10168435; doi:10.21203/rs.3.rs-2852697/v1)
Supplement: Supplement 1 [file NIHPPrs2852697v1-supplement-1.pdf]

Supplementary Files

This is a list of supplementary files associated with this preprint. Click to download.

- SupplementaryMaterialNetworkNPIQ.docx
